# The Transactions of the American Medical Association

**Published:** 1859-05

**Authors:** 


NORTH AMERICAN

MEDICO-CHIRURGICAL REVIEW.

MAY, 1859.

### Analytical and Critical Reviews.

Art I. — *The Transactions of the American Medical Association.* Instituted 1847, vol. xi. Philadelphia: 1858, 8vo., pp. 1027.

To present a satisfactory review of a volume of the size of these Transactions—embracing so wide a range of subjects and of so diversified a character—a review that shall do justice to the authors of the several reports and essays of which the work consists, and, at the same time, offer a careful investigation of the opinions and facts advanced, and a fair exhibit of the actual contribution these transactions furnish towards the advancement of medical science and skill, would demand much more space than can, with propriety, be spared in a periodical journal such as ours. We can do little more than give a general outline of the facts adduced, and the doctrines inculcated in the more important of the papers which compose the volume before us.

We pass over the minutes of the proceedings of the eleventh annual session of the Association, with the simple remark that they are invested with special importance, inasmuch as they exhibit in bold relief the vast and beneficial power the Association is capable of wielding by the exercise simply of the moral influence it holds over the medical profession of our country generally as well as over its individual members.

The proper business of the session was formally opened by an admirable address from the retiring president, Dr. Paul F. Eve. In this address Dr. Eve presents a rapid sketch of the leading objects of the Association, and the extent to which these objects have been thus far carried out through its instrumentality. The entire address was well conceived, and produced a very decided impression by the very eloquent manner in which it was composed and delivered. We entirely agree with Dr. Eve as to the paramount importance of the work to be performed by the American

Medical Association, its capacity to accomplish that work, and the certainty of its ultimate success in the achievement of all the purposes for which it was organized. The Association is now too firmly established and too efficiently organized; it already exercises too powerful and wide-extended a moral influence to allow it to fear the opposition of the most influential persons or local associations within the limits of the profession. We do not hesitate to indorse the declaration of Dr. Eve that the Association has been already "a most active and powerful agent in disseminating useful medical knowledge" throughout this continent; that, in all probability, "no similar institution has ever been more successful than it in carrying out its chief object—the promotion of science."

A careful examination of the eleven volumes of Transactions of the Association will show that they embrace a vast mass of valuable matter, the production of the greater portion of which has been mainly, if not entirely, elicited by the efforts, or, at least, the influence and encouragement, of the Association. Through its agency important facts and principles have been widely disseminated throughout our country, and valuable hints suggested for future investigations, which cannot fail to result, sooner or later, in a profitable addition to our knowledge in every department of medical science. It is very certain, as Dr. Eve remarks, "the American Medical Association has been no failure." It is very true that all the reforms it was instituted to bring about have not been as yet accomplished. Through its instrumentality has neither medical education nor medical literature been elevated to its proper standard in this country; nor do all the papers embraced in its published transactions bear that character for originality of views, depth of research, and excellency of execution which could be desired. Nevertheless, with all these admissions, we maintain that the Association has been eminently successful in "encouraging emulation and concert of action among physicians, and fostering friendly intercourse in the profession." "It has far exceeded," to use the language of Dr. Eve, "the most sanguine expectations, in overcoming all opposition; in creating an admirable code of ethics now adopted everywhere; in organizing state, county, and city societies; in bringing together physicians from the remotest of our immense territory; in awaking the whole profession to its true interests, and in binding us into a common harmonious fraternity;" which, had the Association effected nothing more, should have commanded for it our respect and gratitude. But the good that has been done by and through it exceeds that told above. Through its exertions the general government have been induced to institute measures for preventing the importation of worthless, adulterated, and effete drugs and medicines, and several of the States to adopt laws for the registration of births, marriages, and deaths, while a reference to the professional facts spread out upon the pages of its several volumes of printed transactions will prove how "extensive, varied, and valuable are its contributions to medical science."

The first three of the reports in the volume before us are on Medical Topography and Epidemics for the State of Kentucky, by Dr. W. L. Sutton; for the State of New Jersey, by Dr. Lyndon A. Smith; and for the State of Ohio, by Dr. George Mendenhall.

The first of these reports presents an interesting account of "the jerks," or, as it has been somewhat improperly styled, "epidemic epilepsy." This really curious affection bears a very close resemblance to certain of the convulsive epidemics which prevailed throughout a large portion of the continent of Europe during the middle ages, and which have been so graphically delineated by Hecker.

The disease as it prevailed in Kentucky was consequent upon a great religious excitement which occurred throughout the State, from 1799 to 1805. It was remarkable alike for the extent of the country over which it prevailed, the number of persons of both sexes and of all ages beyond puberty affected by it, and the strangeness and variety of the convulsive and other morbid nervous phenomena by which it was attended. We agree with Dr. Sutton, that the history of these strange affections, which prevail from time to time, with all the leading characteristics of an epidemic, is equally interesting to the physician and to the psychologist.

In this report we have also an account of "milk-sickness, or sick stomach," to which some have given the appellation of malignant fever. The account was drawn up by Dr. James H. Barbour, of Falmouth, Kentucky. Dr. Sutton has added to it some pertinent remarks in reference to the etiology, pathology, and treatment of the disease, derived partly from reading, and partly from oral information communicated by those most conversant with it.

Dr. Sutton does not believe that any relationship, as to cause and effect, exists between the so-called "milk-sickness," in the human subject, and the "trembles," in cattle. In other words, he denies that it is produced by partaking of the milk or flesh of animals laboring under any special or other disease, and includes it among the maladies which have a strictly malarial origin; he believes it to be closely related to the bilious autumnal fever of certain unhealthy localities.

Appended to the report of Dr. Sutton are ten tables. The *first* showing the population of each county of the State, the number of acres each contains, with the proportion improved and unimproved; the number of acres to each inhabitant; the average number that die annually of cholera, dysentery, fevers, scarlatina, and zymotic affections, and the proportion of deaths from each of these diseases to the population in each county. The

*second* table exhibits the annual average mortality of the limestone regions of the State, the average number of deaths in these regions from each of the diseases included in the first table, and the proportion these deaths bear, in each case, to the whole number of deaths from ascertained causes. The *third* table presents the same series of facts in respect to the coal regions. The remaining *seven* tables indicate the color, sex, and age of those who died of cholera, croup, dysentery, continued fevers, fevers generally, scarlatina, and hooping-cough.

The general facts to be derived from a careful analysis of these tables, which appear to have been compiled with great care, are both interesting and important. A similar series of tables for every location in each State of the Union would afford us valuable materials for the investigation of the etiology of those diseases which occur as endemics of particular districts or neighborhoods.

In the report on the epidemic diseases of New Jersey, we find some general conclusions drawn up by its author, Dr. Smith, in respect to the subject of vaccination. To the correctness of nearly all these conclusions we can bear ample testimony.

It is very true, as Dr. Smith remarks, that when there occur, after vaccination, violent inflammation, pain, and swelling, with the production of a large pustule in the arm, and considerable affection of the axillary glands, there is by no means the same certainty of a genuine and permanent infection as when the results of vaccination are of a less intense character. We should never, in fact, pronounce any case in which a genuine *pustule* is produced on the arm, one of true and perfect vaccination. It is proper to remark, however, that our experience has taught us the correctness of the views of most of the German vaccinators, who insist upon the necessity in vaccination of producing a certain intensity of impression upon the system, to insure a complete and enduring protection in the patient against an attack of smallpox. We cannot, therefore, admit the correctness of the position laid down by Dr. Smith, that the production of a "*very small*" vaccine vesicle is in all cases to be preferred.

The report on the epidemics of Ohio is a very concise one. It is chiefly confined to a general consideration of the diseases of the year 1855. It presents no points of prominent interest beyond a notice of the fact that the disposition to bowel affections, which had prevailed throughout the State, to a greater or less extent, and with more or less intensity, from the year 1848 until 1854, had disappeared, and been replaced by a very general prevalence of periodic fevers; confined in 1855 to the months intervening between June and October.

The report of the Committee of Medical Literature follows next in order. It is drawn up by Dr. A. B. Palmer, of Chicago, Illinois. It contains many pertinent and sound observations in reference to the character and amount of the professional literature of the United States, the obstacles which have heretofore prevented its increase and improvement, and the means best adapted for its encouragement in the future. Upon the whole, however, the reporter deals with his subject in general rather than special terms, and, consequently, arrives at no specific and positive conclusions.

Compared with the medical literature of England, France, or Germany, that of our own country is confessedly of small account, whether viewed in reference to its extent or its intrinsic value. When, however, we take into account how short a period, comparatively speaking, has elapsed since the first settlement of these United States; how sparsely populated they still are beyond the immediate limits and neighborhood of a few of our Atlantic and Western States; the nature of the pursuits into which the masses with us are necessarily thrown, and the peculiar influences under which are placed the pioneers in the settlement of a new country, and those who are engaged in bringing it into a condition adapted for the successful development of its resources—all of which circumstances are adverse to purely literary and scientific pursuits,—a full and accurate review of the medical literature of our country, from its earliest colonial history to the present time, will, we are persuaded, exhibit a much more favorable result in respect to the amount and character of the original contributions from the physicians of America than many would seem to be aware. They have not, it is true, produced many works which it will do to compare with those invaluable monographs on special subjects within the range of our science, for which we are indebted to European authors, and but few systematic works in any of the departments of medicine and its collateral sciences, which, in fullness and completeness, are equal to those which come to us from beyond the Atlantic. American physicians have, nevertheless, furnished their quota to the general fund of medical knowledge, and have aided, to an extent equal to their respective opportunities, in the advancement of almost every branch of the healing art, and are now busily occupied in building up a national medical literature, to meet the constantly-increasing demand for it on the part of the medical profession of the country.

In investigating the medical literature of the United States, we shall find a very great scarcity of independent treatises by American authors. None, in fact, of any imposing pretensions previously to the last fifteen or twenty years. The most important and valuable contributions of our physicians, with very few exceptions, are to be sought for in the pages of the leading professional journals, which, from an early period of our national existence, have constituted, in a great measure, the library of the

American physician; certainly of the majority of those the most extensively engaged in practice out of our larger cities. The importance, even at the present day, of well-conducted medical journals, to the profession throughout the country, cannot be too strongly insisted upon; these publications present an easy medium of communication, through which may be effected a ready interchange of the facts and observations collected, and the conclusions arrived at by the physicians of every portion of the United States and of Europe, and the wide diffusion of these contributions to the general fund of medical knowledge, at short intervals and at so trifling an expense as to render the labors and experience of each member of the profession, both here and abroad, promptly available for the instruction of all. Medical journals form, also, convenient reservoirs for the reception and preservation of a mass of facts, which, in their isolated form, are of comparatively little value, but which, nevertheless, are to be ranked among the materials essential for the repair of the errors and for supplying the defects of existing medical theories and modes of practice.

To give to our professional journals the usefulness we have claimed for them, to render them faithful and ready mediums of intelligence—channels for the prompt dissemination of the improvements made in the different departments of medicine; to enable them to become a correct and vivid reflection of the actual knowledge and opinions of the entire profession, requires that they should be conducted by able, enlightened, and independent editors—skillful to detect whatever of merit the various articles submitted for publication may possess, and possessed of the moral courage that will enable them to reject the valueless and insert the good, irrespective of any considerations connected with the name, standing, and connections of their respective authors.

While a strict censorship should be exercised by the conductor of a medical journal over the matter and the tone of whatever articles are admitted to its pages, so that wild speculation, imperfectly observed facts, and observations of very doubtful accuracy shall be carefully excluded, as well as even the shadow of misrepresentation or injustice toward the labors of others, a very great degree of leniency should be observed in respect to mere inaccuracies or inelegancy of style, such, at least, as do not involve any gross violation of grammar, or vulgarity of manner, or lead to a degree of obscurity calculated to confuse or mislead the reader. From the degree of influence they exercise over the opinions and practices of a large and interesting portion of our profession in this country, medical journals must be ranked as among the most important means for the promotion of domestic medical literature. In every point of view in which they can be correctly contemplated, they deserve our hearty support when ably and honestly conducted.

The following is a brief résumé of some of the leading positions advanced in the report:—

"The periodical literature of the United States is regarded as possessing great abundance, variety, richness, and general excellence, and though still possessing defects, is constantly improving. Many of the contributions are of great weight and value, indicating an enterprising and industrious profession. Serious defects are regarded as existing in the review department, arising mainly from the fact that the income of the journals will not justify pecuniary disbursements for literary labor, and editors necessarily engaged in other pursuits cannot command the time, if all possessed the ability to do the work thoroughly and well. A few well-supported journals, in place of the many but illy sustained, might tend to correct this evil; but the multiplicity of local journals is considered as peculiarly beneficial, by collecting from a greater variety of sources a larger number of facts, and developing the powers of a larger number of writers. The interests of this part of our literature demand a more prompt and liberal pecuniary support.

"The number of original American medical works is increasing, and their character is improving, and in some respects, in practical utility, they will not suffer in comparison with those of Europe; yet serious imperfections exist, and decided improvements are demanded. Great and permanent improvements in medical, as in general literature, must be gradual, depending more upon the advancement of education, of taste, and intelligence, than upon any specific measures which may be adopted. Still, various particular measures, such as the frequent writing of medical theses during professional pupilage, and keeping systematic records of cases when in practice, would do much in hastening on improvement. But, for the greatest perfection of our literature, we must wait the fuller development of our country, and for those changes of time and circumstances which shall produce a larger number of devoted savans and scholars, placing them in situations where a variety of absorbing pursuits will not prevent the concentration of great talents upon a comparatively limited range of subjects.

"Respecting the reprint of foreign works, it is held that while the free circulation of the best class of these books among us increases the knowledge and improves the taste of the masses of the profession, it does not interfere with the production of the higher order of original works, and that the moral obligation of our government to join with Great Britain in the enactment of an international copyright law, is by no means clearly established."

There is great truth in the following remarks:—

"Our locality has an influence upon our character, our science, our literature, and our claims upon others. We are upon a new continent, have a vast territory, are in neighborhood of imperfect governments. We have much to subdue, to regulate, to establish. We have great internal seas and extended rivers, which, together with our railroads, invite to travel, to change, to commerce. Our people are from diversified sources. They have relations with all parts of the world, and possess extended knowledge of its affairs. The material and practical are thus pressed upon us. Our youth, as a nation, has been sufficiently referred to.

"With all these practical duties and affairs, we have not yet had time to perfect many great scholars. Amid the multiplicity of objects of thought and action which surround us, we have not yet found time for the minuter study of any limited number. This country is not now the place for the development of the individual in limited and special directions. But it is the place for giving individuals a wide range of knowledge, though less minutely and perfectly, and it is also the place for the diffusion of knowledge among the whole people, and, as previously stated, it is above all others the place for making the knowledge of others, the results of others' labors practically useful, of showing the diversified applications and the practical value of scientific truths. In this we excel. Here, in the great economy of improvement, is our sphere. This is our place in the great brotherhood of science and art. This is our position in the republic of letters. We have, then, in this economy a fixed place, and a manifest destiny. That place we must maintain, and that destiny fulfill. We may be charged with appropriating the materials, the results of the labors of others. We do, and we must thus appropriate. Our necessities and our destiny demand it. We must have knowledge cheaply, freely, unobstructedly. The *masses* of our profession and the *people* must have knowledge. In the great family of enlightened nations we are the last born. Our scientific and literary wants are beyond our own capacity to supply. In our youth we must be sustained. The mother that bore us, our elder brothers, must help us. We are already making a return, particularly in the department of practical results. Scarcely a patient in Europe submits his flesh to the surgeon's knife, but has the keen anguish averted by our own discovered anæsthesia. Perpetual incense of praise and thanksgiving ascends to God and America for this great boon. In our very first lispings we taught the people of Europe to avert the lightning's stroke, and in later, but still youthful accents, we have instructed them to make that lightning do their bidding. We are, then, in our youth making some returns to the present Europe, but, in our manhood, we shall make greater returns to the future humanity. In our growing youth we may appropriate more than we yield. In our full maturity we shall yield more than we appropriate. We may be borrowing of the present, but we are paying at least our annual interest, and the full principal and interest, compounded at that, shall be returned, followed by heavy advances to the future humanity."

The report on Medical Education follows. It is by Dr. James R. Wood, of New York.

If all the physicians throughout the United States would agree among themselves as to the nature and extent of the knowledge and other qualifications necessary to be possessed by such as claim admission into the ranks of the medical profession to entitle them to an entrance, and adopt some uniform test by which the possession of the requisite qualifications could in every case be certainly determined; if they would then uniformly withhold all professional intercourse and countenance from such as assumed the name and office of physician without having been subjected to the test, and been proved by it worthy, there would be no difficulty in respect to the schools, for they would be at once forced to bring up their course of instruction to the standard demanded by the profession. So long, however, as we admit the right of each medical school to fix the nature and extent of the professional education necessary to obtain admission for its pupils into our profession, to confer upon any one whom they please the authority to practice medicine, it will be in vain to attempt any decided reform in medical education in this country, or to establish any uniformity in respect to the qualifications of our physicians throughout a single State—leaving out of question the entire Union. The regulation of the terms of admission into its ranks must be undertaken by the medical profession itself, and so soon as its members shall become alive to a true sense of their own interests, they will find themselves fully qualified for the task of defining the line of separation between the legitimate practitioner of medicine, and they who assume the office without due preparation for the faithful performance of its duties—a line that would not fail, ultimately, to be recognized by the public, and to become an effectual barrier against the invasion of the proper rights and interests of the profession by the ignorant and unprincipled.

From the recommendation embodied in the report before us, in respect to a convention of delegates from the medical colleges of the United States, "for the purpose of devising a uniform system of medical education," we can scarcely anticipate any very favorable results. The conclusions of Dr. Wood as to the plan of medical instruction demanded, are as follows. So far as the plan is set forth, we have no especial objections to urge against it:—

"*First.* Primary medical schools should be encouraged, but, as office instruction will continue to be sought for by students, practitioners should either give them the necessary advantages of demonstrations, illustrations and recitations, or if not prepared to do so, they should refer them to such primary schools, or medical men, as will give them proper instruction.

"*Second.* The number of professorships should not be less than seven, viz.: a professor of anatomy and microscopy, physiology and pathology, chemistry, surgery, practical medicine, obstetrics, and materia medica.

"*Third.* There should be but one term annually, which should commence *about* the first of October, and close with the March following, thus lengthening the term to six months. The commencement of the term, in October, should be uniform in all the colleges throughout the country. During the session there should never be more than four lectures given daily.

"*Fourth.* The qualifications for graduation, in addition to those now required in the schools, should be a liberal primary education, and attendance upon a course of clinical instructions in a regularly organized hospital."

The next report in order is on Spontaneous Umbilical Hemorrhage of the New-born, by Dr. J. Foster Jenkins, of New York State. The subject is a most interesting one, and which has only of late received the attention it justly demands. The report of Dr. Jenkins will be found to embrace a very good analysis of all the facts found upon record calculated to throw light upon the history of umbilical hemorrhage in the new-born, its pathology and treatment, with the addition of a series of observations derived from his own experience and that of several of his medical friends. The leading particulars of all the cases that have come to the knowledge of the reporter, are presented in tabular form.

In respect to treatment, Dr. Jenkins considers the safest and most reliable to be that recommended by P. Dubois, of Paris; that is, to transfix, with two harelip-needles, (one being passed horizontally and the other perpendicularly,) the integuments at the base of the umbilicus, and then to pass several times, around the needles, in figure of 8, a waxed thread. The needles may be removed on the fourth or fifth day, but the eschar should be allowed to separate spontaneously without any attempt being made to hasten its detachment. Although the ligature *en masse*, as thus described, will occasionally fail, in common with all other means, to arrest the discharge of blood in cases of spontaneous hemorrhage from the umbilicus of young infants, it should nevertheless be resorted to without delay, from the fact that it is more to be depended on than anything else that has been employed in these cases.

The report on the Influence of Marriages of Consanguinity upon Offspring, by Dr. S. M. Bemiss, of Louisville, Kentucky, recommends itself to the candid consideration of every class of the community. If it can be satisfactorily shown that upon the offspring of intermarriages between blood relations are liable to be entailed mental or physical imperfections or diseases, it then becomes an all-important question, not merely with the philanthropist, but with every enlightened statesman, what are the means to be adopted in order to prevent the marriage of the consanguineous? To determine with certainty whether any, and if any, what deleterious influence is exercised upon the offspring by such marriages is, however, a somewhat difficult task. To do so it is necessary to ascertain the number of offspring born, with and without any physical or mental deficiency or disease, in each community, and in each class of that community, during a long series of years, first of parents between whom there is no relationship of blood, and secondly, of parents in whom such relationship does exist, taking into account, in both cases, the occupations, habits, conditions, and hereditary predisposition of the people of each community and class. By then comparing together the results in the two cases, some definite conclusion in respect to the influence of marriages of consanguinity upon the children born of them, may be arrived at.

So far as the report goes, it presents a mass of valuable materials for the solution of the problem submitted to the committee from whom it emanates. Dr. Bemiss has collected the facts bearing upon the condition of 3942 children, namely, 1509 males and 1411 females born of 833 marriages of consanguinity. Of these children, 1134 were defective, 145 deaf and dumb, 85 blind, 308 idiotic, 38 insane, 60 epileptic, 300 scrofulous, 98 deformed; 833 died young, and 53 were sterile, thus showing that only 838 of these offspring of marriages between blood relations were free from some defect or disease.

We return our thanks to Dr. Bemiss for what he has done in elucidation of the question under consideration, and we trust that his attention, in common with that of other physicians, will continue to be directed to the subject, with a view to the accumulation of a mass of direct and comparative statistics adapted to settle definitely the influence which the marriage of the consanguineous produce upon the offspring born of such marriage; whether a single intermarriage between blood relations is sufficient to entail upon the children a defective physical and mental organization, and a proneness to certain diseases, or whether this baneful influence upon the offspring is not rather the result of repeated intermarriages between the descendants of those related by blood.

A short report follows on the Functions of the Cerebellum, by Dr. E. Andrews, of Chicago, Illinois. Though confessedly imperfect, it is still of a character to command attention, and will repay an attentive perusal.

The theory advanced by the author is, that the lateral lobes of the cerebellum, which are, properly speaking, the posterior, preside over the muscles of the posterior group, while the median lobe exerts its influence upon those of the anterior group. In support of this theory, the following propositions are advanced, the truth of which Dr. Andrews endeavors to establish by reference to an anatomical examination of the cerebellum in different warm-blooded animals:—

"1. In the warm-blooded animals, the median lobe, or vermiform process of the cerebellum, varies in size directly as the bulk and power of the anterior group of muscles.

"2. The lateral lobes vary in like manner, as the power of the posterior group of muscles, subject, however, to certain variations hereafter to be mentioned."

In reference to these exceptions, Dr. Andrews remarks, that while the lateral lobes have a certain correspondence with the posterior groups of muscles, they have a no less striking ratio with the size of the hemispheres of the brain, and the rank of the animal in the scale of intelligence.

"There are, therefore," according to Dr. Andrews, "two conditions governing the lateral lobes as to size, which may be illustrated by three propositions:—

"1. If the animal have large complex hemispheres of the brain, and, at the same time, very powerful posterior muscles, then both these conditions unite in demanding large lateral lobes of the cerebellum, and these organs, in fact, attain, under such circumstances, their maximum development."

This is illustrated by a reference to the cerebellum of man and the dolphin.

"2. If the hemispheres of the brain be of a low grade, but the posterior extremities be still powerful, then the deficiency in one condition is balanced by the excess in the other, and the animal will have lateral lobes bearing a moderate ratio with the median."

Illustrated by a reference to the cerebellum of the kangaroo, agouti, wild rabbit, and porcupine.

"3. If the animal have cerebral hemispheres of decidedly inferior development, and at the same time posterior limbs which are feebler than the anterior, then both the conditions will coincide to reduce the lateral lobes to a bulk less than the median."

Illustrated by a reference to the cerebellum of birds, moles, bats, of the sloth, etc.

The facts deduced from the examination of the cerebellum in a large number of the mammalia leads, Dr. Andrews believes, to the following conclusions:—

"1. The division of the cerebellum which we have been considering, is traceable from the reptiles up to man.

"2. The typical position of the lateral lobes is posterior to the median.

"3. The median lobe has some function connected with voluntary motion, and its influence is expended chiefly upon the muscles of the anterior half of the body.

"4. The lateral lobes exert a similar influence over the muscles of the posterior half of the body, and they are also in some way connected with the mental functions, inasmuch as they are developed, to a certain extent, in direct ratio with the intelligence of the animal.

"5. The *nature* of the influence exerted by the cerebellum upon the muscles is not very clear. Although it may be true, that through it the mind co-ordinates the muscular action, yet that is *not* true which is stated by Carpenter and others upon the subject, viz., that the size of the cerebellum is in a direct ratio with the number and variety of co-ordinated movements which the animal is capable of exercising. Within the limits already explained, the size of the cerebellum is *directly as the quantity and power of muscular fibre to be moved*, with no regard whatever to the simplicity or complexity of their combinations."

Dr. Andrews admits that the foregoing conclusions indicate simply *the direction* in which the facts of comparative anatomy point, but are not in themselves decisive, unless they shall be confirmed by a new and full examination of the facts derivable from experiment and from pathology.

In the next report, that of Dr. Mark Stephenson, of New York, on the Treatment best adapted to each Variety of Cataract, we find nothing new—nothing with which every well-instructed ophthalmic surgeon is not, or at least ought not to be perfectly familiar; but little, in fact, of importance, that is not more clearly and accurately laid down in all the leading treatises on surgery. So far as the report presents us with the personal experience of its author, it possesses, of course, a certain amount of interest. And yet in making up our judgment as to the operation to be adopted in each case that presents itself, we are to be guided, not simply by the kind of cataract that is present, but also the kind of eye in which the cataract exists, the age, general health and constitutional tendencies, and the particular influences under which the patient to be operated on is placed.

The succeeding report is on the Medical Jurisprudence of Insanity. It is drawn up by Dr. C. B. Coventry, of Utica, New York. The subject of which it treats is in all its bearings a most important, and, at the same time, most difficult one. Its interest is not confined to the physician and criminal jurist and judge, but extends to the community at large. It is, in fact, assuming every year a character of deeper, wider, graver import in its bearings upon the safety, in person and property, of each citizen, and in its reference to the humane and faithful administration of the laws. The plea of insanity is at present urged in almost every case to shield from conviction, not merely such as are arraigned in our courts of justice for criminal violence and homicide, but such also as stand accused of crimes against property: it is essential, therefore, that the true line of discrimination should be drawn between those who assume a character of insanity as a cloak to screen them from the punishment of their evil deeds, those who though actually deranged in mind are nevertheless capable of judging between right and wrong, and such as, by reason of their mental derangement are rendered irresponsible agents, and should not be treated as criminals, but rather as fit subjects for one of those asylums in which, while they are restrained from doing mischief, an effort may be made to restore them to "their right mind."

In the report before us, we are presented with a general sketch of the pathology, classification, and tests of insanity; a brief consideration of the plea of insanity in criminal cases; the kind and degree of mental derangement that should warrant the depriving of an individual of his liberty or the management of his estate, that should invalidate a contract or will made by him, or avoid responsibility for crime; a word is said in respect to feigned insanity, and some judicious remarks are appended in respect to the testimony of skilled witnesses in courts of justice. Under each of these heads there will be found much to instruct.

We have next, from the pen of Dr. E. Jarvis, of Massachusetts, a report on the Law of Registration of Births, Marriages, and Deaths. It is replete with judicious suggestions calculated to render the registration more perfect, and to extend it to other States than the few by which it has been already adopted. To the report is appended a nosology for the mortality of 1850, prepared from De Bow's list of diseases for the use of the Census Department of the United States; a nosological classification; the classification and nomenclature of diseases according to Dr. Wm. Farr, of London; and a list exhibiting the principal causes of disease, classified and arranged in the order in which they have been considered proper for statistical purposes.

The report which follows is on the Nervous System in Febrile Diseases, and on the classification of Diseases by the Nervous System. It is a somewhat elaborate and certainly most able inquiry into the agency of the nervous system in the production of those groups of morbid phenomena which, in consequence of their almost invariable concurrence, have been erected by the nosologist into separate and distinct forms of disease.

The author of the report, Dr. H. F. Campbell, of Georgia, is already favorably known to our readers as the first who pointed out the existence of an excito-secretory nervous function, and by his deductions in reference to the influence of the several portions of the nervous system in the production of the various pathological conditions of the animal organism.

Setting out with the general proposition that "all morbid actions are more or less influenced in their manifestations by aberrated nervous action," he attempts to show that "as in the nervous system we recognize two grand departments, viz.: First, the cerebro-spinal system, all the normal actions of which are subject to cessation and interruption; and secondly, the ganglionic system, all the normal actions of which are of a *continuous* and *uninterrupted* character, so in the manifestations of febrile disease we distinctly recognize two grand distinguishing characteristics, respectively typifying the normal actions of these two symptoms of nerves. Thus a character of *paroxysm* obtains in certain cases, while a character of *continuousness* as plainly marks the others." And again, that—

"As in the cerebro-spinal system we find that its normal action pertains almost exclusively to sensation and to motion, with only a secondary and comparatively remote influence (which we have termed excito-secretory) upon nutrition and secretion, while in the normal action of the *ganglionic system* the entire function is known to preside over nutrition and the secretions, so in *paroxysmal fevers* do we find intense pain, modified sensation, and symptoms allying them to neuralgia and convulsive diseases very prominent, while in *continued fevers*, modified nutrition and altered secretion are the marked and most prominent characteristics."

We cannot pretend to follow Dr. Campbell in his illustration of the foregoing propositions, nor in his application of them to the establishment of a correct neuropathic system of pathology. Much less can we spare time and space—even had we the inclination—to enter upon a critical examination of the truth of his general propositions, or the correctness of all the views he has based upon them in respect to the characteristics of diseases and their proper classification.

Much of the report is necessarily occupied with a retrospective view of the opinions held by distinguished pathologists in respect to the immediate subject of inquiry, or which have a more or less direct bearing upon it. From these Dr. Campbell has drawn valuable materials adapted to illustrate and confirm the theory he has advanced, as the one which most satisfactorily interprets the complex phenomena of fever, and traces these to their true dynamic sources, so as to establish upon the latter a proper basis of classification.

It would not be correct to say that Dr. Campbell has succeeded in building up a full and satisfactory pathology of the febrile affections, it being to these his attention has been chiefly directed in the present report. All that can be truly conceded to him is the credit, and this, we may remark, is no trifling one, of having rendered probable the position that it is upon some one or other portion of the nervous system that morbific agents act primarily, and, according as these agents affect chiefly or entirely some portion of the cerebro-spinal, or the ganglionic system of nerves, will be the nature of the disturbance produced by them in the organs, and tissues, and fluids of the body, and the character of the morbid phenomena thence resulting. In a word, the credit of having rendered it more than probable that the several portions of the nervous system have, each of them, a special agency in determining the direction and kind of morbid action that shall occur in any given case, when an individual is exposed to either of the causes of disease.

Even in this limited sense the labors of Dr. Campbell will afford us no little aid in the establishment of a true system of pathology, one that will be able to reconcile some of the contending and apparently inconsistent truths which have been adduced in support of opposite views of the nature and cause of the morbid states of the solids and fluids of the animal organism, of the vices of structure and disturbances of function observed in disease.

The following "tabular arrangement" of diseases presented by Dr. Campbell, will convey to the reader some general idea of the pathological views advanced by him:—

"NEUROSES.—Diseases in which any portion of the nervous system may be recognized as the *primary dynamic source of either their functional manifestations, or their structural changes.*

"CLASS 1. CEREBRO-SPINAL NEUROSES.—All paroxysmal. The cerebro-spinal system primarily affected. Produced by causes generally of an atmospheric origin, introduced into the blood. May also be excited by any irritation, whether peripheral or central, as urethral irritation, wounds, furuncles, etc., and as in case of injuries of the nervous centres.

"*Order* 1. CEREBRO-SPINAL APYREXIÆ.—Little or no aberration in the chemical changes going on in the tissues. Altered nutrition and secretion rarely manifested. The characteristic of this order is the *absence of excito-secretory action* as a general thing, though it is occasionally manifested in each one of them.

"Genus 1. *Neuralgiæ.*—Affecting the sensory portion of the cerebro-spinal system. These have been variously divided in accordance with their location, character, etc. etc.

"Genus 2. *Spasmi.*—Affecting the motory or excito-motory portion of the cerebro-spinal system. These are specifically arranged, also, in accordance with their characters and locations, as—

"Species 1. *General Convulsions.*

*a.* Tetanus,

*b.* Epilepsy, etc.

"Species 2. *Partial Convulsive Affections.*

*a.* Pertussis.

*b.* Spasmodic Croup.

*c.* Spasmodic Asthma.

} More frequently marked by excito-secretory action in one form or another, than the rest of this genus.

"*Order* 2. CEREBRO-SPINAL PYREXIÆ, OR CEREBRO-SPINAL FEVERS.—The characteristics of this order is, that they manifest strikingly the law of *periodical excito-secretory* action in every possible form, as by periodic aberrations in the secretions, circulation, and nutrition in the chemical changes, and, consequently, in the changes of animal temperature. They are *par excellence* the *excito-secretory neuroses*—allied to the cerebro-spinal apyrexiæ by pain and convulsive symptoms.

"Genus 1. *Cerebro-Spinal Fevers of Many Paroxysms.*—Generally endemic. The paroxysms or exacerbations continue to return at stated intervals, unless prevented by appropriate therapeutic measures. The effect of each paroxysm is to produce or to increase congestions in one or more of the organs, by excito-secretory action; by frequent repetition these congestions become true engorgements. Both the congestions and engorgements permanently derange the functions of the organs in which they occur. Hæmatopoiesis is interrupted and deranged, the patient becoming in time œdematous and pale. Did we consult these local congestions, these fevers might be arranged as would respect the popular, but rather superficial and objectionable, distinction of brain fever, lung fever, bilious fever, intestinal, gastric, etc. A marked characteristic of the genus is, also, their tendency to hebdomadal periodic relapses.

"Species 1. *Intermittent Fever.*—*Complete* subsidence of febrile symptoms during the intervals between *paroxysms.*

"*Varieties* 1. Quotidian.

2. Tertian.

3. Quartan, etc.

"Species 2. *Remittent Fever.*—*Partial* subsidence of febrile symptoms during the intervals between *exacerbations.*

*Varieties* 1. Quotidian.
2. Tertian.
3. Quartan, etc.

"APPENDIX TO THIS GENUS.—These may be either intermittent or remittent, or they may be epidemic or sporadic.

1. Pneumonia.
2. Pleuro-pneumonia.
3. Influenza, pharyngitis, etc.
4. Cholera infantum.
5. Dysentery, etc.

"Genus 2. *Cerebro-Spinal Fevers of a Single Paroxysm.*—Nearly always epidemic. Closely allied to malarial fever, and to the apyrexiæ, by pain, convulsive symptoms, etc. Far transcend the malarial forms in the destructive changes wrought in the blood, and in the tissues of the organs. Length of paroxysm, from twelve to seventy-two hours. The paroxysm does not repeat itself after having entirely subsided. Often marked by remissions.

"Species 1. *Yellow Fever.*

*Varieties* unknown, or not fully determined.

"Species 2. *Dengue Fever.*

*Varieties* unknown, or not fully determined.

"CLASS II. GANGLIONIC OR SECRETORY NEUROSES.—All of a continued or non-paroxysmal character. All refer *directly* to the ganglionic system as the dynamic source of the phenomena—all of them, whether apyrexic or pyrexic, are produced by causes which can generally be recognized as being of a specific nature. Some of them are contagious, others not so. They are either epidemic or endemic.

"*Order* 1. GANGLIONIC APYREXIÆ.—The interstitial chemical changes giving rise to increased animal temperature, not manifested.

"Species 1. *Simple Diarrhœa.*—Sporadic and endemic.

"Species 2. *Asiatic Cholera.*—Epidemic.

"Order 2. GANGLIONIC PYREXIÆ, or GANGLIONIC FEVERS.—As their name indicates, the characteristics of fever are well marked. Interstitial chemical changes abundant, resulting in high temperature. Each one of the order is self-limited in its duration.

"Genus 1. *Typhoidal Fevers.*

"Species 1. *Typhoid Fever.*

*Varieties* 1. Thoracic form.
2. Abdominal form, etc.

"Species 2. *Typhus Fever.*

*Varieties* not arranged.

"Genus 2. *Exanthematous Fevers.*

*Examples* 1. Scarlatina.
2. Rubeola.
3. Variola, etc. etc."

We have next a well written, carefully digested, and able report on Moral Insanity in its relations to Medical Jurisprudence, by Dr. D. Meridith Reese, of New York.

In examining in its several bearings the subject of his report, with a view to deduce its true medico-legal importance, Dr. Reese discusses the following questions:—

"I. What is 'moral insanity,' as defined by those who have introduced the term into our medical nomenclature, and who seek to connect it with our jurisprudence, and obtain its recognition by our tribunals of morals and law?

"II. Wherein does 'moral insanity' differ from 'moral depravity,' in any case in which 'intellectual insanity' does not coexist?

"III. If insanity, whether intellectual or moral, be the result of physical disease in the brain, either functional or structural, are not the distinctions into partial or monomaniacal insanity, into mental and moral, wholly fabulous and visionary?

"IV. In deciding all questions of responsibility and punishability, ought not the only distinction to be between *sane* and *insane?* The responsibility and 'punishability' of the insane is prohibited by every code of law and morals, whether divine or human. The *sane*, and none else, are responsible and amenable to punishment, while the contrary may be infallibly predicated of the *insane*, whether the insanity be physical, intellectual, or moral."

Moral insanity, in the sense in which the term is generally employed and understood by those who insist upon the recognition of such a form of mental disease, may be defined "a perverted state of the natural feelings, affections, inclinations, temper, the moral disposition and impulses existing without any illusion or hallucination, and with a more or less weakened, impaired, or uncultivated state of the intellectual faculties. The perversion of the feelings, propensities, and moral sense being to such an extent as to subjugate the will and hurry the individual, upon the spur of appetite, passion, or interest, by an irresistible impulse to the commission of crime, at which his judgment and his conscience, provided they were left free to act, would revolt."

Does such a state of things ever exist? If facts oblige us to reply in the affirmative, then we have certainly a form of moral insanity, the presence of which ought to be a sufficient plea for unaccountability—unpunishability. Dr. Reese denies the existence of any such separate perversion of the moral feelings and propensities—of the moral sense. According to him, the supposition of moral insanity coexisting with intellectual sanity, is a fable, unless *moral depravity* and *intellectual* purity can govern in the same mind at the same time, which he properly denounces as a mental and moral absurdity. He contends that—

"1st. Insanity is a *mental* phenomena, symptomatic of a *physical* disease, having its seat in the *brain*, and hence can neither be *intellectual* nor *moral* exclusively, but necessarily involves both *thought* and *feeling*, for the reason that the *mind* is the *source*, and the *brain* is the *organ* of both.

"2d. No alleged insanity, which is not preceded or accompanied by *positive disease in the brain*, of which the physical signs are cognizable and demonstrable, should be regarded by our courts of civil or criminal jurisdiction, as justifying the judgment, either of *incapacity* or *irresponsibility*.

"3d. When any disease of the brain is proved to exist, and a perversion or alienation of the moral or intellectual functions of the mind is present, while the subject is unconscious of any change in his mental or moral status, in all such cases insanity exists to an extent demanding impunity from the penalty of any law, and the protection of society, as well as the safety of the insane subject, requires that he be committed to the custody and remedial influence of an asylum or hospital until his restoration to health and a sound mind is satisfactorily established by competent and adequate proof.

"4th. All alleged 'moral insanity,' if unaccompanied by the preceding and simultaneous proofs of disease of the brain by characteristic symptoms, should be regarded as *moral depravity*, for which, as well as for its consequences, the party should be held accountable to the law.

"5th. No metaphysical or psychological theory of 'irresistibility,' or the loss of 'moral freedom,' should receive the countenance of our profession, in the absence of unequivocal evidence of physical disease involving the brain.

"6th. Neither 'delusion,' 'illusion,' 'hallucination,' 'systematic design,' 'knowledge of good from evil, or right from wrong, or that the act was lawful or unlawful, or contrary to the laws of God and nature,' or 'impassivity,' or 'hereditary taint,' can be safely relied on in deciding upon responsibility or irresponsibility, the presence or absence of disease in the *brain* being the only true criterion of sanity or insanity.

"7th. In all cases of acquittal from crime, on proof of insanity, perpetual restraint upon liberty during life, by committal to an asylum, or until full proof of entire recovery, should be secured by *statute law*. The confinement of insane convicts should be in a separate and distinct institution, nor should they ever be admitted to the asylums or hospitals for other insane patients.

"8th. Medical psychology, especially in its relations to judicial inquiries on the subject of insanity, should be made an integral part of medical education, and clinical teaching should be introduced into every asylum for the insane, as a measure of public policy, the duty to devolve upon the superintendent of each.

"9th. Until a general provision is secured for the education of all students in medical psychology, no physician who has not special qualifications, both by study and practice in this department, should consent to give testimony in cases of alleged insanity, unless after consultation and concurrence with an acknowledged expert. The reputation of the profession will else be jeoparded, if not compromised and destroyed, by indiscreet opinions, which the courts are obliged to overrule, or which are often notoriously disapproved."

Much good sense and sound practical wisdom seem to us to pervade the whole of the foregoing conclusions. To admit that "moral insanity," in the very loose sense in which the term is used by many, is a sufficient cause for the acquittal of an individual charged with the commission of crime, would be, in our opinion, to open a wide door for the escape of the morally depraved from the just punishment of their evil deeds, and which, without having any tendency to further the cause of true humanity, will rather promote than diminish the prevalence of crime.

A report on Stomatitis Materna is presented by Dr. D. L. McGugin, of Iowa. This disease, which has so frequently been observed in females during pregnancy, or the period of lactation, especially the latter, and which has claimed the attention of many acute observers who have given to us very full and interesting accounts of it, with their views of its nature and causes, though prevailing unquestionably more frequently in certain countries and districts of country than in others, is, we suspect, however, of occasional occurrence wherever there are pregnant women and nursing mothers. That it is not formally noticed by certain physicians of large practice in particular localities, is no evidence that they had never met with it, but rather that they looked upon it in the light of an affection of the buccal mucous membrane, to which others are equally as liable as pregnant and nursing females, when exposed to certain morbific causes. Dr. McGugin insists that the disease is *sui generis*, attacking only the female during pregnancy or lactation; when existing independent of either of these conditions, it being always the "continuation of an invasion first made" during one or other of them. In support of this opinion he has, however, adduced no satisfactory evidence.

In all the cases of stomatitis materna that have occurred under the notice of Dr. McGugin, the patients were of a strumous habit, presenting unequivocal indications of the presence of scrofula or of a tuberculous diathesis. The predisposition is, in all probability, due in every instance to a debilitated or broken-down constitution.

The exciting causes are, according to the author of the report before us, *pregnancy* and *lactation*, but prominently the latter. He supposes, and with some show of truth, that the disease is the immediate result of "some error or perversion of digestion and nutritive assimilation," and that pregnancy and lactation, especially the latter of these conditions, act as its exciting causes, by perverting or interfering with the due performance of these functions.

"The change," he says, "takes place in stomatitis, in the blood; and the process of nutrition, which is the prominent feature in the sex, is interfered with, for other pressing demands must first be answered. The organized material being withdrawn or applied to another purpose, the tissues to which it rightfully belongs suffer from the want, and become impoverished. Softening of these structures must follow, and to this again will be added ulceration as a consequence of innervation. The circulation being feeble in structures thus deprived of heir nutrition, will become congested, and ulcerative inflammation follows."

This, no doubt, is all very correct; but we suspect that in most instances we have, as predisposing and exciting causes of the disease, certain debilitating agents besides the constant drain from the system caused by suckling. Such as cold and dampness, malaria, deficient or unnutritious food, grief and anxiety, want of out-door exercise, etc.

The treatment recommended by Dr. McGugin is one adapted to increase or supply the nutrient principles of the blood, to prevent all unnecessary waste of the strength and vigor, and to relieve the lesions of the mouth and fauces. Daily out-door exercise, nutritious diet, porter, ale, and malt beer, tepid bathing, dry air, cleanliness, and cheerfulness; in extreme and lingering cases, weaning the child. As internal remedies, nitrate of silver, iodide of iron, hydriodate and chlorate of potassa, vegetable tonics, hypophosphite of soda, subnitrate of bismuth, etc. etc.

On the True Position and Value of Operative Surgery as a therapeutic agent, is the theme of the report next in order. It is drawn up by Dr. J. B. Flint, of Louisville, Kentucky, and places the subject on a just basis.

The late Dr. Physick was accustomed to remark, when commencing the operative department of his course of lectures on surgery, that the skill of the true surgeon is not based upon the number and boldness of his operations, but rather upon the frequency with which he is enabled, having a due regard at the same time for the best interests of his patients, to dispense with the use of the knife. It is true that cases occasionally occur in which a surgical operation, involving even positive and permanent maiming of the body, is, no doubt, absolutely necessary in order to preserve the life, or reinstate the health of the patients. Surgical operations are, nevertheless, to be viewed as the lowest and least desirable of all our therapeutic agents. As remarked by Dr. Flint, "in regard to almost every instance of its exercise, the place of operative surgery is among the 'last resorts' of practice. Not until we have exhausted all other means of defending life or member against the attacks of disease, are we warranted in resorting to those terrific operations which seldom save without pain or danger somewhat commensurate with their expected benefits."

Dr. Flint's denunciation of what he denominates "*dramatic*" surgery, the so-called "exploital" and "heroic," is at once manly and well timed. We fully agree with him that such operations, got up with a view to attract attention, and gain for the performer credit as a "bold operator," "should be scrutinized very carefully before they are reckoned among the legitimate agencies of the art, or allowed to become the criteria of professional ability or merit." "The fascinations of 'dramatic surgery' are dangerous to professional morality, and mischievous to society, and we should endeavor to replace them by just and rational views of the operative proceedings of our art."

An interesting description of a New Method of Preserving Membranous Pathological Specimens is next given by Dr. R. Arnold, of Savannah, Georgia. The method of Dr. Arnold is to attach the specimens to the surface of a plate of window-glass, and, when dry, covering them with a coating of white varnish.

A short letter follows from Dr. E. D. Fenner, of New Orleans, embracing some remarks on the non-efficiency of quarantine as a means of preventing the occurrence of yellow fever.

We now come to the two prize essays, the one, by Dr. Austin Flint, of Buffalo, New York, on "The Clinical Study of the Heart-sounds in health and disease;" the second, by Dr. Montrose A. Pallen, of St. Louis, Missouri, on "Vision, and some of its anomalies, as revealed by the ophthalmoscope."

The essay of Dr. Flint is marked throughout by that closeness of observation and cautiousness of deduction for which all his contributions to the medical sciences are distinguished. We sincerely regret that we are prevented from giving to the paper that consideration which the importance of the subject itself, and the able manner in which it is discussed, would seem to require. Having already transcended the limits assigned us for the length of our review, we can do nothing else than present the summary of conclusions relating to the heart-sounds in disease, as drawn up by the author of the report.

"1. The two elements which compose the first sounds of the heart, viz., the valvular element, and the element of impulsion, may be affected conjointly but unequally, or one element may be affected exclusively of the other, and either may be extinguished while the other remains.

"2. Of the two sounds which unite to form the valvular element, viz., the mitral and the tricuspid, the former may be abnormally diminished in intensity, or extinguished, while the latter continues, and its intensity is in some instances greater than in health. When this is the case, injury or destruction of the mitral valves is indicated. An abnormal intensity of the tricuspid sound, under these circumstances, denotes hypertrophy of the right ventricle, and there will probably be found, at the same time, exaggeration of the *pulmonary* sound of the heart.

"3. To determine whether weakness or extinction of the mitral valvular sound be due to injury or destruction of the mitral valves, or to diminished muscular power of the heart's action, this sound is to be compared with the element of impulsion. If the latter element, over the apex of the heart, be well marked, while the valvular element without the left nipple be feeble or wanting, it is to be inferred that the mitral valves are more or less seriously damaged. This inference, however, is not to be drawn if it be suspected that the mitral sound is not appreciable or imperfectly heard, in consequence of the intensity, roughness, or diffusion of a coexisting murmur.

"4. On the normal intensity of the mitral valvular sound, when a murmur is present, referable to the mitral orifice, may be predicated the opinion that the abnormal changes, giving rise to the murmur, have not as yet compromised seriously the integrity or function of the mitral valves.

"5. When morbid conditions exist, organic or inorganic, which intensify the *first* sound of the heart in hypertrophy and functional disorder, the intensity of both elements of this sound is increased, but the element of impulsion is exaggerated disproportionately to the valvular element. So, also, when the muscular power of the heart is weakened by any cause, the auricular valves remaining unaffected, the element of impulsion is enfeebled more than the valvular element, and the latter is sometimes lost when the former is still appreciable. Both elements, however, may be extinguished by causes which weaken the muscular power of the heart, while the *second* sound continued to be heard in different situations.

"6. If, from any cause, the *first* sound be weakened so as to extinguish the element of impulsion, but not the valvular element, this sound, in duration and quality, resembles the second sound. When this is observed over the apex of the heart, where the element of impulsion is most marked in health, it does not follow that the heart is dilated. Weakness of the systolic movement is alone indicated, and this change in the duration and quality of the *first* sound at the point stated, occurs in cases of great hypertrophy as well as dilatation, if the muscular power of the organ be sufficiently impaired. The same change may occur when the muscular power of the heart is impaired by fatty substitution, softening, and by dynamic causes.

"7. Abnormal intensity of the element of impulsion occurs when the muscular action of the heart is increased from any cause. Mere abnormal intensity may not indicate more than the increased action due to functional excitement, but if the *first* sound be notably prolonged and dull, characters which depend on the element of impulsion, hypertrophy is indicated. The absence of these characters, however, is no evidence against the existence of hypertrophy, since the muscular power of the organ may be diminished to an extent to weaken and even extinguish the element of impulsion when the organ is greatly hypertrophied.

"8. The *second* sound of the heart being constituted by a valvular element alone, is not subject to modifications which are practically important, except as regards its intensity and the degree of valvular quality. It may be exaggerated, weakened, or extinguished, and the valvular quality more or less marked.

"9. The *second* sound emanating not from one source, but consisting of an aortic and pulmonary sound, which are distinguishable from each other in certain situations in health, the abnormal modifications may relate to either, exclusive of the other. The aortic and pulmonary sounds may be separately increased or diminished in intensity, and each may be extinguished, the other remaining.

"10. Extinction of the aortic sound, the pulmonary sound not being suppressed, denotes destruction or very serious injury of the aortic valves. This conclusion is corroborated by the presence of a murmur referable to the aortic orifice.

"11. Abnormal weakness of the aortic sound denotes injury to the aortic valves, especially if an aortic murmur be present, provided that this weakness be not attributable to mitral contraction or regurgitation, or to impair muscular power of the heart. The presence or absence of mitral disease may generally be determined by the presence or absence of a murmur referable to the mitral orifice; and to determine whether the weakened aortic sound be due to impaired muscular power of the heart, this sound may be compared with the pulmonary sound, which, in health, is feebler than the aortic. Evidence of enfeebled action of the heart is also obtained by observing the *first* sound. The *second* sound of the heart, however, is comparatively much less affected by the various conditions which involve impairment of the muscular power of the organ, than the *first* sound.

"12. On the normal intensity of the aortic sound may be predicated an opinion that the aortic valves are sound. When this sound is found to retain its normal intensity, in connection with a diastolic murmur, the fact renders it probable that the murmur is produced by the direct current of blood through the mitral orifice, and not by aortic regurgitation. When the normal intensity of this sound continues in connection with a systolic murmur referable to the aortic orifice, it shows that, although a lesion exists in this situation, it has not, as yet, led to serious change of the valves.

"13. Exaggerated intensity of the aortic sound occurs in cases of hypertrophy affecting the left ventricle, so long as the muscular power of the organ is not impaired. This alone, however, is not a sign of hypertrophy, for the sound is also exaggerated when, from any cause, the power of the heart is increased.

"14. Abnormal modifications of the pulmonary sound relate, so far as their importance practically is concerned, to an increased intensity of this sound. When it is found to be relatively more intense than the aortic sound, it denotes either that it is positively exaggerated, or that the aortic sound is abnormally weakened, or both these conditions may exist. If the aortic sound be not abnormally weakened, greater relative intensity of the pulmonary sound is evidence of hypertrophy of the right ventricle. In connection with this exaggeration, the tricuspid portion of the valvular element of the first sound will be likely to be intensified. Whether a greater relative intensity of the pulmonary sound be due to a positive exaggeration, or to diminished intensity of the aortic sound, or to both combined, it is generally observed in cases of mitral disease, attended by either obstruction or regurgitation. This rule, however, is not without exceptions."

In noticing the essay by Dr. Pallen, on Vision, and Certain of its Anomalies, we scarcely know what language to employ. As an able digest of our present knowledge in regard to the anatomy and physiology of the parts concerned in vision, it does credit to its author, and would be an appropriate and valuable chapter for a treatise on physiology, or introductory to a work on ophthalmic medicine. Dr. Pallen has collected, with no little industry and skill, the observations and opinions of the most authoritative of those who have in modern times investigated with the greatest success the structure and functions of the eye and its appendages; but, in respect to neither of these subjects has he presented anything original—any new facts, or new deductions based upon the facts of others. We confess that we cannot see a single claim which the author of the essay before us can base upon it to entitle him to a prize of one hundred dollars.

It may be that Dr. Pallen may claim the credit of having presented a more full and exact description of the ophthalmoscopical phenomena of the diseased eye than is to be met with in any of the works which are within the reach of the generality of practitioners; but even if this should be admitted, the essay before us can scarcely lay claim to the kind and degree of merit we ought to expect in the papers honored with the prizes of the American Medical Association.

